# The impact of the use of personal-protective-equipment on the minimization of effects of exposure to pesticides among farm-workers in India

**DOI:** 10.3389/fpubh.2023.1075448

**Published:** 2023-03-08

**Authors:** Summaiya Lari, Praveen Yamagani, Arun Pandiyan, Janardhan Vanka, Mohan Naidu, Balakrishnan Senthil Kumar, Babban Jee, Padmaja R. Jonnalagadda

**Affiliations:** ^1^ICMR-National Institute of Nutrition, Hyderabad, Telangana, India; ^2^Department of Biochemistry, Acharya Nagarjuna University, Guntur, Andhra Pradesh, India; ^3^Department of Health Research, Ministry of Health and Family Welfare, Government of India, New Delhi, India

**Keywords:** pesticides, farm-workers, occupational exposure, personal-protective-equipment, risk assessment, cholinesterase

## Abstract

**Introduction:**

Although excessive use of pesticides and unsafe agricultural practices may contribute to numerous intoxications, the role of PPE (personal-protective-equipment) in the minimization of toxicological effects due to pesticide exposure has not been addressed so far. The present study aimed to assess the impact of the use of PPE on the minimization of effects of exposure to pesticides among farm-workers.

**Methods:**

A community-based follow-up study with questionnaire-based survey and field observations was undertaken among farm-workers (*n* = 180) of Rangareddy district, Telangana, India. Biomarkers of exposure such as cholinesterase activity, inflammatory markers (TNF-α, IL-1β, IL-6, cortisol, and hs-C reactive protein), nutrients (vitamins A, E), liver function (total protein and A/G ratio, AST and ALT levels) were investigated in the laboratory by following the standard protocols.

**Results:**

Farm-workers who had a mean farming exposure of 18 years of and who neither followed safe pesticide handling practices nor used PPE and also showed reluctance to obey good agricultural practices (GAPs). Inhibition of AChE (acetylcholine esterase) with increased inflammation was found among farm-workers as compared to their respective normal values when they have not used PPE. Linear regression statistical analysis revealed a profound effect on inhibition in the AChE activity and various inflammatory markers with the increase in the duration of pesticide exposure. Further, there was no effect of the duration of pesticide exposure on the levels of vitamins A, E, ALT, AST, total protein, and A/G ratio. Further, intervention studies carried out on the use of PPE provided (commercially available and cost-effective) for 90 days showed a significant reduction in the biomarker levels (*p* < 0.01).

**Conclusion:**

This study demonstrated the importance of the use of PPE during pesticide applications and other agricultural tasks to minimize pesticide-associated adverse health effects.

## 1. Introduction

Pesticide formulations containing organic minerals, and synthetic, or natural chemical substances, are used extensively to enhance crop productivity. Although their use leads to increased yield of agricultural crops to meet the demands of global food supply, their use has become a matter of public health concern as it is associated with acute morbidities and mortalities (1). There are several reports available on the association between chronic exposure to pesticides and metabolic disorders, such as diabetes, obesity, and other cardiovascular, and neurodegenerative diseases which are characterized by enhanced oxidative stress and inflammation (2). Therefore, the role of exposure and the resulting risk assessment has become extremely significant, particularly for occupationally exposed groups.

Occupational exposure to pesticides in the agricultural workplace occurs during the preparation (mixing and loading) and application (spraying) of pesticides, with this exposure being several orders of magnitude higher than that occurs among the general population who are indirectly exposed either to traces of pesticides through their contamination in the food/water or through environmental exposure (3). Reports are also available on the deficiencies in agricultural hygiene such as unsafe and non-preventive pesticide handling practices including the careless disposal of empty pesticide containers, limited use of personal-protective-equipment (PPE), deficiencies in safety training practices, etc., among the Indian farming community who get directly exposed to pesticides (4, 5). Further, the use of mixtures of pesticides is also related to a higher incidence of pesticide poisonings and deaths. The poverty and malnutrition in combination with the multiple exposures to a wide range of pesticides of moderately and highly hazardous toxicity categories also makes them more vulnerable (6).

High-level exposure to pesticides is known to produce a variety of biochemical alterations, some of which may even be responsible for the adverse biological effects in humans (7). Though all of them may necessarily not lead to clinically recognizable symptoms but can be used as biomarkers of exposure or effect (8), which provides a critical tool for epidemiological studies examining the complex interactions between toxicants and human health (7). Of the various, Acetylcholinesterase (AChE), oxidative stress, free radicals, inflammatory markers, and liver function measures are vital (9–12). In addition, the nutrients also exert ameliorating effects against pesticide-induced toxicity (13, 14).

Moreover, there is a lack of scientific evidence with defective large-scale surveillance data to estimate the magnitude of the problem and the significant association between pesticide use and chronic adverse health effects in developing countries (15). Since pesticides enter the body through dermal, oral, and inhalation routes; PPE may minimize the farmers' risk of exposure to pesticide poisoning (16). The lack of information on the use of protective measures and exposure data inhibits an accurate assessment of the extent of their exposure (17). Unfortunately, in low-income countries (LICs), recommended PPE is not widely used by farm workers for several reasons like unawareness, inaccessibility, un-affordability, discomfort caused due to heat and humidity, considered unnecessary, and the belief that it causes illness (18–20). To our knowledge, very few studies have examined the effect of the use of PPE among farm workers exposed to health hazardous pesticides, even if available, such studies were inadequate because of small sample size, pesticide exposure time from handling, mixing, loading till spraying was not reported, none or few potential confounders were only taken into account, full body protection was not tested, relative comparison with other PPE standard was not made and other potential methodological issues (21–25). Therefore, the present study aimed to evaluate biochemical alterations due to long-term exposure to pesticides among farm-workers of the Rangareddy district of Telangana, India using AChE activity and various other hematological/biochemical parameters as biomarkers of effect. The present study implicates that the use of PPE may play a critical role in minimizing the exposure and thereby reducing the likely adverse health effects among farm workers in their interest as they are backbone of the nation in terms of food security.

## 2. Materials and methods

### 2.1. Study design and subjects

It is a community-based follow-up study conducted in four identified villages of Rangareddy district in Telangana, India. Considering the prevalence of 84.14 with 95% confidence interval, 80% power, and 20% effect size, a total of 180 farm-workers (60 subjects each from paddy, vegetable, and commercial crop cultivators such as cotton) were considered for their participation in the study (26). Both male and female farm-workers (farm men and farm women) with an age group of 18 to 50 years who are continuously engaged in agricultural activities were included; while individuals with cardiovascular, diabetics, hyper/hypo tension, dermatological allergy, other allergic fungal infections, pregnant women, liver disease/damage, alcoholic/viral hepatitis, metastasis, obstructive jaundice, using pyridostigmine drug, who underwent cardiac surgeries and those who were unwilling to comply with study conditions were excluded.

**Figure 1:**
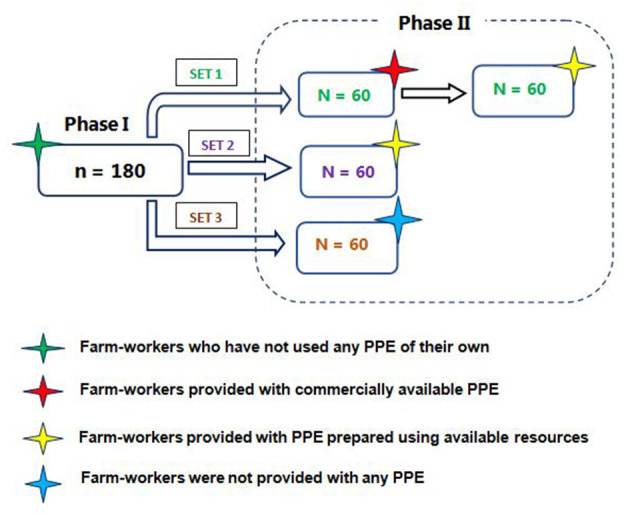
Distribution of subjects in first and second phase of the study.

First phase of the study included the farm-workers (*n* = 180) during their regular farming activities who have neither used PPE in any form of their own nor provided with any type of PPE and the samples were collected for the purpose of estimation of hematological/biochemical parameters. Further, second-phase intervention studies were carried out for the exposed farm-workers to assess the impact of the use of PPE provided (coverall, gloves, boots, goggles, and masks) on the pesticides exposure. In the second phase of the study, both male and female farm-workers (*n* = 180) were divided into three sets. The first set of farm workers (*n* = 60) were randomly selected and were provided with both commercially available and cost-effective PPE, while the second set were provided with only cost-effective PPE and the third set were not provided/used any PPE (Figure 1). The commercially available PPE were as per norms of European Food Safety Authority guidelines and the Pesticide Handler Exposure Database (27, 28) which includes a Tychem 'C' category III cover-all (DuPont™); while the cost-effective PPE include cover-all designed and prepared by using available resources. Both types of PPE were provided for free of cost along with a safety splash goggle; a cup type respirator; a pair of nitrile gloves and a pair of PVC gumboot (Usha Fire, DuPont supplier, Hyderabad, India).

During the period of 90 days of their participation in the cultivation of respective crops, they were advised to wear the PPE provided to them over their regular farm clothes whenever they are involved in handling the pesticides or engaged in any other agricultural activities, followed by the collection of samples. Further, they were instructed not to handle pesticides and abstain from exposure for seven days before commencing the second phase of the study, since according to World Health Organization “an acute pesticide poisoning is any illness or health effect resulting from suspected or confirmed exposure to a pesticide within 48 h” (29). They were also monitored continuously during the follow-up phase of the study to adopt GAPs and adequately use the provided PPE.

### 2.2. Ethical clearance and consent

To avoid any potential bias, the farm-workers were made clear that the study is being conducted only in the interest of academic research. Written informed consent was also obtained from them and they were also explained that they are at the liberty to withdraw from their participation at any given point of the study period without any fine or penalty. The names of the farm-workers were replaced with specific codes to ensure confidentiality. The study protocol was reviewed and approved by the ethical committee of the Indian Council of Medical Research—National Institute of Nutrition, Hyderabad, India (REF NIN Protocol Number 11/I/2016).

### 2.3. Questionnaire data and field observations

A five-page questionnaire which was pre-tested among 30 subjects before its administration containing both closed- and open-ended questions consisting of 172 variables was administered to farm-workers (*n* = 180) to collect information on the socio-demographic characteristics; particulars of exposure to pesticides while engaged in various agricultural activities and handling the pesticide formulations; personal habits; knowledge and practices while handling the pesticides; precautions followed; and details on the morbidity/mortality symptoms associated due to exposure during/after handling of pesticides (Supplementary File). Further, the information from each farm-worker on each separate occasion was also recorded using standardized field data sheets which includes types and quantities of active ingredients handled during the day; number of times and total duration of exposure; types of work clothing (shirt/T-shirt, cotton cloth fabric, length of sleeves, trousers, shoe, scarf, if any) used; incidences of spills and leakages, etc. Observations such as their re-entry into the treated fields, walking direction during spraying, incidental contaminations, and events such as using damaged equipment, talking, smoking, or eating/drinking during handling of pesticides were also noted. Data on meteorological parameters of maximum and minimum temperature (°C), relative humidity (%), wind velocity (km/h) and direction were also recorded using digital anemometer (LM 8010, Lutron Electronic, Taiwan) each time on the day of samples collection followed by the spraying of pesticides at every point of application for the respective crop in the treated fields.

### 2.4. Blood sample collection

Approximately 8 mL of blood sample was collected through venipuncture in Becton Dickinson (BD) vacutainer tubes containing heparin/EDTA as an anticoagulant under aseptic conditions from each farm-worker. The samples collected were transported from the field to the laboratory under chilled conditions using gel packs. In the laboratory, the plasma and serum were separated from whole blood by centrifugation (1S-R Multifuge, Heraeus, Thermo Scientific) at 3,000 rpm for 5 min and stored at −20°C in decontaminated and labeled eppendorf vials till further analyzed.

### 2.5. Estimation of acetylcholine esterase activity

Changes in cholinesterase activity in serum due to exposure to pesticides were estimated as per the kit protocol obtained from Bharat Enterprise, Hyderabad, India, by adopting the Kinetic Propionyl-thiocholine method using semi-autoanalyzer (MERCK Microlab 300) (30). About 1 mL of working cholinesterase buffer solution which was prepared freshly was taken in a cuvette to which 20 μL of serum sample was added followed by measuring the activity of each unknown sample obtained from the instrument by reading the absorbance at 405 nm at 15 and 45 s. The assay was found to be linear up to 8,000 U/L.

### 2.6. Estimation of various hematological/biochemical parameters

Enzyme-linked immune-sorbent assay (ELISA) kit method was used for the quantitative determination of various hematological/biochemical parameters as per the instructions given in the kit protocol and performance characteristics were done for each assay (Table 1). C-reactive protein (CRP), interleukin-6 (IL-6), interleukin-1 beta (IL-1β), tumor necrosis factor-alpha (TNF-α), human aspartate aminotransferase (also known as AST or SGOT) and alanine aminotransferase (also known as ALT or SGPT) were estimated in serum samples using kit procured from Diagnostic Biochem, Canada and Krishgen Biosystems, Mumbai, India. The readings were measured using a micro-well plate reader (Biotek Synergy H1 Hybrid) with Gen5 2.01 software at 450 nm. While, the estimation of cortisol, total protein, vitamins A and E in plasma samples was done using assay kit protocol (Kinesis Dx, USA) and analyzed by following the biuret method (31, 32) by measuring the readings at 550 nm within 60 min using a semi-auto analyzer (Merck Microlab 300). Further, albumin was determined in the plasma samples using an assay kit (Kinesis Dx, USA) based on BCG (bromocresol green) method (33), wherein the absorbance was measured against the blank at 630 nm using a micro-well plate reader (Biotek Synergy H1 Hybrid) with Gen5 2.01 software. The globulin levels in g dL^−1^ were calculated by subtracting albumin (g dL^−1^) values from total protein (g dL^−1^); subsequently, albumin/globulin (A/G) ratio was also calculated.

**Table 1:** Performance characteristics of various hematological/ biochemical parameter assay kits.

**Hematological/biochemical parameter**	**Assay range (sensitivity)**	**Correlation co-efficient (*R*^2^)**	**Recovery range (%)**
CRP (ng mL^−1^)	10–10,000 (10)	0.9924	92–109
IL-6 (pg mL^−1^)	3.125–200 (1)	0.9957	93–102
IL-1β (pg mL^−1^)	3.91–125 (1)	0.9905	80–114
TNF-α (pg mL^−1^)	7.81–500 (1)	0.9936	96–111
AST (U/L)	0.5–100 (0.251)	0.9995	87–117
ALT (U/L)	0.5–100 (0.23)	0.9996	91–112
Cortisol (pmol mL^−1^)	75–1,200 (7.186)	0.9943	80–114
Total protein (g dL^−1^)	1–15 (1.95)	0.9995	75–99
Vitamin A (μg mL^−1^)	15–240 (1.32)	0.9926	86–111
Vitamin E (μg mL^−1^)	3–48 (0.31)	0.9989	79–104
Albumin (g dL^−1^)	1–7.0 (1)	0.9969	82–110

### 2.7. Statistical analysis

The data were analyzed using the Statistical Package for Social Sciences (SPSS) version 28.0.0.0 (IBM SPSS Statistics). The descriptive variables were represented as mean (standard deviation), frequency and percentages for farm-workers who have not used PPE (*n* = 180). Pearson chi-square analysis was carried out to assess the association between demographic particulars and the various knowledge, attitude, and practice parameters. Linear regression analysis was also performed to determine the effect of the duration of pesticide exposure on levels of multiple biomarkers studied among the farm-workers when they have not used PPE and not followed GAPs. Further, based on the normality assumptions, either a paired *t*-test or Wilcoxon signed ranks tests were performed to assess the significance of the AChE levels and the hematological/biochemical parameters observed before and after the use of commercially available and cost-effective PPE provided to them. Further, the associations were also studied with a 95% confidence interval (CI), equal variances were not assumed, and statistical significance was considered at *p* < 0.05 and *p* < 0.01.

## 3. Results

### 3.1. Characteristics of farm-workers

Detailed personal characteristics of the farm-workers studied have been reported elsewhere (34), while in the present investigation data for selected farm-workers (*n* = 180) is presented. The mean age (years) of farm men (*n* = 113, 62%) was found to be 39.8 years while it was 33.7 years for farm women (*n* = 67, 38%). Most of the farm-workers (92%) reported that their houses were located away from the farms while 68% work as agricultural or other laborers and 32% have their own agricultural farms. The average number of years of involvement in the farming activities reported by the farm-workers was found to be 18 years and the average extent of land holdings calculated was 3.88 acres. Information collected using a pre-tested questionnaire and other field observations are presented (Table 2).

**Table 2:** Basic demographic background pesticide-related awareness, attitude and practice among the surveyed farm-workers (*n* = 180).

**Parameters**	***n* (%) of farm-workers**	**Chi score**	***p*** **-value**
Gender		3.471	0.176
Male	113 (62)		
Female	67 (38)		
Education		57.041	0.001^**^
Illiterate	45 (25)		
Read and write	79 (44)		
Primary (1st−5th)	35 (20)		
Secondary (6th−10th)	15 (8)		
Inter	2 (1)		
Degree and above	4 (2)		
Farming experiences	Mean 18.01 years		
Exposure history		20.410	0.026^*^
<15 years	73 (41)		
15–22 years	54 (30)		
>22 years	53 (29)		
Main Occupation		1.880	0.930
Agriculture	58 (32)		
Tenant Cultivation	48 (27)		
Agriculture labor	65 (36)		
Other labor	9 (5)		
Personal habits		5.631	0.002^**^
Non-vegetarians	176 (98)		
Lacto-ovo-vegetarians	4 (2)		
Smoking cigarettes or beedis^#^	117 (65)	3.616	0.164
Consuming alcohol^#^	94 (52)	7.793	0.020^*^
Use of personal protective equipment (PPE)		5.668	0.001^**^
Yes	0		
No	178 (99)		
Any other (handkerchief / towel)	2 (1)		
Mixing of pesticides		2.264	0.322
Bare handed	159 (88)		
With gloves on	0 (0)		
With aid of wooden stick or metal rod	21 (12)		
Pesticides storage		1.394	0.845
In the farm in a separate shed	116 (64)		
In the house in a separate room	43 (24)		
In the house along with other items	21 (12)		
Disposal of the empty containers of pesticides		1.965	0.568
In the agricultural fields	71 (39)		
In the canal/passage of the agricultural fields	23 (13)		
In the open/barren fields	29 (16)		
In the dumping ground where the waste material is dumped	25 (14)		
Sell as scrap	32 (18)		

# Multiple responses allowed.

Statistical significance was considered at

* *p* < 0.05

and

** *p* < 0.01.

**Figure 2:**
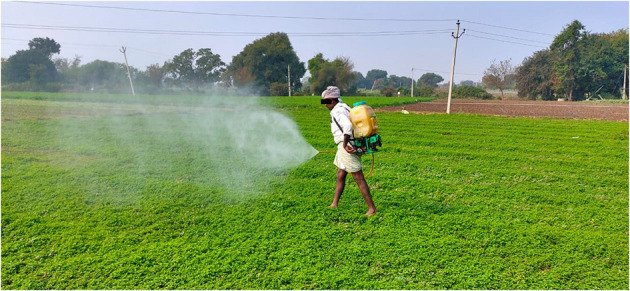
Farm-worker involved in the spraying of pesticides using backpack pump without following safety protocols.

Further, 94% of farm-workers were using hand spray using a knapsack or backpack sprays with a hand-pressurized pump. It was noted that the sprayings were done by positioning the lance in front of the farm-workers when they walk forward in different directions in the treated field areas (Figure 2). Besides, they also admitted the fact that the equipment used by them was nearly ten years old with some defects such as damage or leakage from spray tanks through nozzles or connecting pipes. Further, the majority of the farm-workers (98%) were unable to precisely indicate the exact names or quantities of pesticide formulations that they have used or using, as they depend on the retailers for the type/quantity of pesticides to be sprayed onto the crops cultivated by them which however, depends/varies as per the crop cultivated, the intensity of the pest infestation and the areas to be treated. Hence, the details on the types of pesticides used by the farm-workers were collected from the nearby retailers and the *Krishi Vigyan Kendras* of agricultural extension centers located in the identified villages of each area studied (Table 3).

**Table 3:** Commonly used pesticides in the studied area and the long-term adverse effects.

Class: Active ingredients (WHO classification^*^)	**Trade name used in the area**	**Long-term adverse effects**
OPs: Acephate (II)	Acemain, Acestar, Startin	AChE inhibition in humans; nausea, dizziness, confusion, respiratory paralysis and death (35)
OPs: Profenofos (II)	Orax, Profit	AChE inhibition in humans; compulsive licking, abnormal gait, salivation, lacrimation, impaired respiration, ataxia, impaired reflexes, tremors, and decreased arousal, rearing, and motor activity (36)
OPs: Chlorpyrifos (II)	Dursban, Hilban	Cholinesterase inhibition in humans; intermediate syndrome or organophosphate-induced delayed polyneuropathy; emesis, respiratory failure, tachycardia, kidney injury, and seizure (37)
OPs: Monocrotophos (Ib)	Monocil	Nausea, vomiting, diarrhea, abdominal cramps, headache, dizziness, eye pain, blurred vision, constriction or dilation of the pupils, tears, salivation, sweating, confusion, slurred speech, loss of reflexes, weakness, fatigue, involuntary muscle contractions, twitching, tremors of the tongue or eyelids, involuntary defecation or urination, psychosis, irregular heartbeat, unconsciousness, convulsions and coma; respiratory failure or cardiac arrest may cause death (38).
OPs: Phorate (Ia)	Timet, Gulkal	Kidney damage; bradycardia, salivation, lacrimation, diaphoresis, vomiting, diarrhea, urination, miosis, tachycardia, hypertension, mydriasis, and muscle cramps, CNS depression, agitation, confusion, delirium, coma, seizures, ventricular dysrhythmias, metabolic acidosis, pancreatitis, and hyperglycemia (39)
OPs: Quinalphos (II)	Dhanulux	Decrease in sperm motility and total epididymal sperm count and an increase in sperm abnormality; weakness and fatigue (40)
Neonicotinoid class: Imidacloprid (II)	Confidor	Fatigue and paralysis, dizziness, drowsiness, disorientation, coma, sweating, dilated pupils, tachycardia, and hypertension which may lead to coronary spasm and cardiac ischemia and risk of arrhythmia (41)
Emamectin Benzoate (NL)	Proclaim, Benzer	Induced DNA damage and apoptosis; generate ROS; genotoxic effect on human lung cells (42)
CMs: Mancozeb (U)	M-45, Mancozeb	Thyroid disease and neural tube defects in newborns (43, 44)
CMs: Carbosulfan (II)	Marshal	Cholinesterase enzyme inhibition in humans; liver pathology (40)
Benzimidazole: Carbendazim (U)	Prem	Testicular toxicity (45)
SPs (II): Cypermethrin, Lambda-cyhalothrin, Delta-methrin	Ninja, Cypermethrin, etc.	Neurotoxic and gastrointestinal effects in humans; neuronal degeneration and increase in glial cells in brain, and disorganization of hepatic laminae, increase in sinusoid, and necrosis of hepatocytes in liver (animal) (46, 47)
OPs: Triazophos (Ib) + SPs: Deltamethrin (II)	Sarpanch, Tiger	AChE inhibition; tremor, muscle contraction or spasticity and dyspnea (animal) (48)

* WHO, World health organization acute toxicity hazard class (38); Ia, Extremely hazardous; Ib, Highly hazardous; II, Moderately hazardous; U, Unlikely to present acute hazard in normal use; NL, Not Listed.

OPs, Organophosphorus pesticides; CMs, Carbamates; SPs, Synthetic Pyrethroids pesticides.

**Figure 3:**
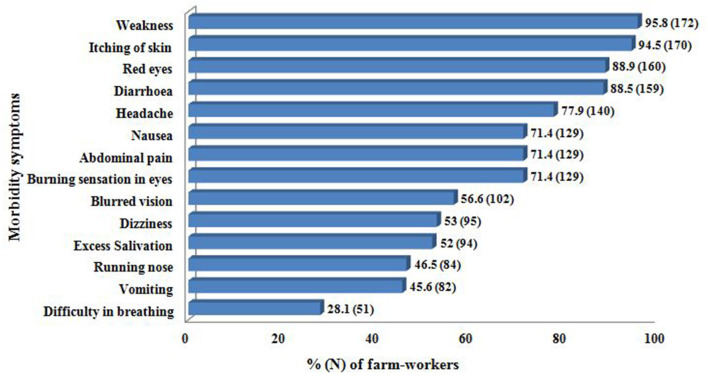
Pesticide poisoning self-reported morbidity symptoms by farm-workers (*n* = 180).

The information on the details of meteorological conditions indicated high temperature between 27.8 and 41°C (mean 35.28°C), relative humidity as 7.2% to 65.4% (mean 34.36%) with an average southwest wind velocity of 8.91 km/h throughout the duration of their participation in the respective fields. It was also observed that most of them do not follow the wind direction when spraying the pesticide formulations. Further, most of the farm men (62%) wore long trousers, long sleeved cotton shirt, and rubber shoes; while rest (38%) wore short sleeved shirt, *lungi* (sarong) and slippers. On the other hand, all the farm women wore *saree* (Indian traditional wear) and slippers with a long sleeved shirt over the *saree* in order not only to cover themselves but also to protect themselves from exposure to heat. Further, some of the farm men also explained that they do spray pesticide formulations with bare body occasionally to avoid the heat stress. Further, 99% of farm-workers were not using PPE of their own while handling the pesticides. The reasons indicated by them for not using the same varied, as some of them (44%) stated that it is inconvenient to wear, while for 51% it was inaccessible, and others (5%) felt suffocation. It was further found that none of the participants have received any professional official training on pesticide handling or GAPs. In addition, majority of them also explained self-reported associated morbidity symptoms immediately after spraying the pesticides (Figure 3).

### 3.2. Effect of duration of pesticides exposure on various levels of biomarkers

**Figure 4:**
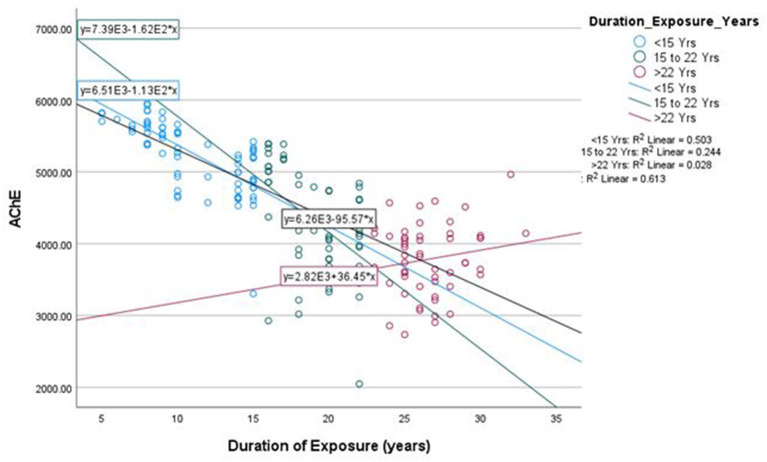
Linear regression between duration of exposure and AChE activity among exposed farm-workers (*n* = 180).

**Figure 5:**
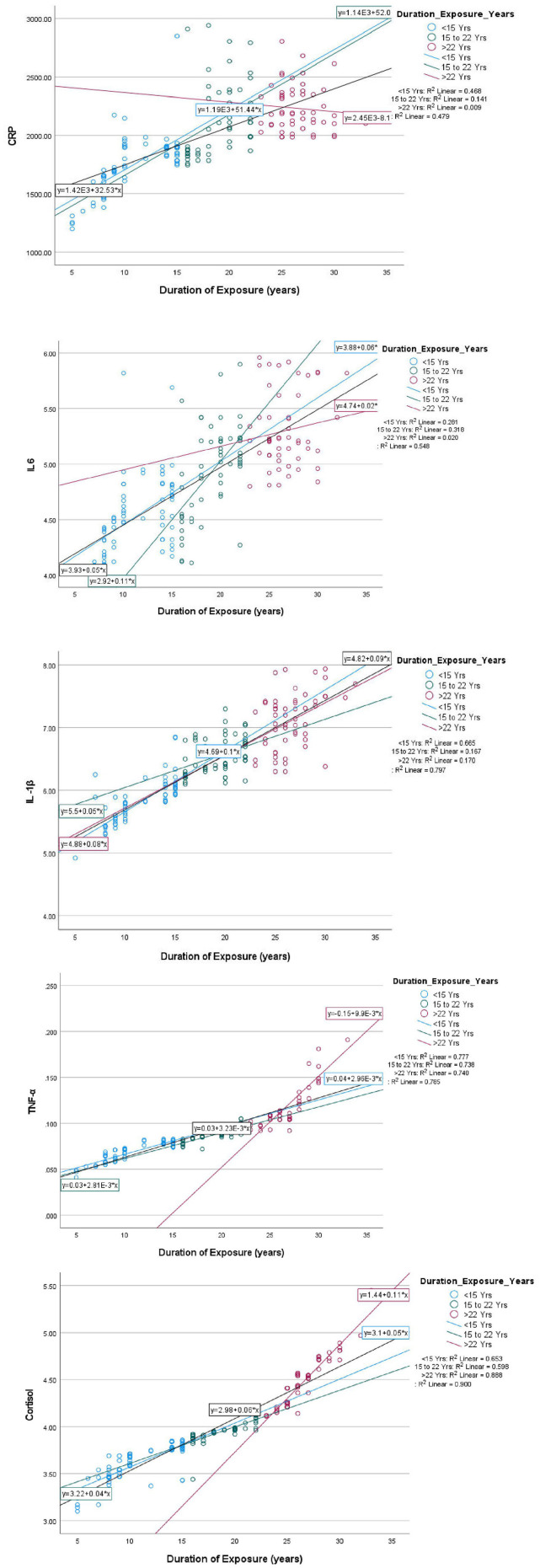
Linear regression between duration of exposure and levels of various inflammatory biomarkers (CRP, IL-6, IL1-β, TNF-α and cortisol) among exposed farm-workers (*n* = 180).

The mean duration of pesticides exposure of farm-workers (*n* = 180) who had not followed safety protocols and/or adopted GAPs was 18 years (Table 2). The duration of exposure corresponds to the direct handling of pesticides by the farm-workers, which includes preparation (mixing and loading of pesticide formulations), application (spraying of pesticide formulations), and their involvement in other agricultural activities such as sowing, watering, thrashing, cutting, harvesting, weeding, cleaning, and maintenance of spraying equipment, etc. The linear regression analysis performed on the effect of duration of pesticides exposure on AChE activity and levels of various inflammatory biomarkers among exposed farm-workers without using PPE revealed that with every 1 year of increase in the duration of exposure, there was an inhibition in the AChE activity by 95.57 U/L (R^2^ = 0.613) (Figure 4). Further, the duration of pesticides exposure also exerts profound effect on the various inflammatory markers, as with every 1 year increase in duration of exposure, there was an increase in CRP levels by 32.53 ng mL^−1^ (R^2^ = 0.479), IL-6 levels by 0.05 ng mL^−1^ (R^2^ = 0.548), IL-1β levels by 0.09 ng mL^−1^ (R^2^ = 0.797), TNF-α levels by 0.0033 pg mL^−1^ (R^2^ = 0.785) and cortisol levels by 0.06 pmol mL^−1^ (R^2^ = 0.9) among the exposed farm-workers (Figure 5). However, no such effects were found on the levels of vitamins A, E, ALT, AST, total protein and A/G ratio.

### 3.3. Effect of non-use/use of PPE on acetylcholinesterase activity

The results showed a reduction in AChE activity among all the farm-workers when they have not used PPE as compared to the normal range (>4,900). Further, the inhibition of AChE was not only relatively less and their values were also above the normal range when they have used either commercially available or cost-effective PPE provided to them. A significant difference (*p* < 0.01) was observed in the AChE activity when they have used either type of PPE versus when PPE was not used by them (Table 4).

**Table 4:** Acetylcholinesterase activity (U/L) among different groups of subjects.

**Sr. No**.	**Groups of subjects**	**Mean**	**SD**	**Range**	***p-*** **value**
1	Without using PPE (*n =* 60)	4,432	854	2,904–5,849	0.000^**^
	After using only commercially available PPE (*n =* 60)	5,564	692	4,102–6,737	
2	Without using PPE (*n =* 60)	4,825	785	3,019–6,951	0.000^**^
	After using only cost-effective PPE (*n =* 60)	5,419	741	3,893–6,659	
3	Without using PPE (*n =* 60)	4,432	854	2,904–5,849	0.000^**^
	After using both commercially available and cost-effective PPE (*n =* 60)	5,261	606	3,936–6,530

SD, standard deviation; Statistical significance among the different groups of subjects was considered at

** *p* < 0.01.

### 3.4. Effect of non-use/use of PPE on various hematological/biochemical parameters

To assess the impact of pesticides exposure, the plasma/serum samples were subjected for the estimation of various hematological and biochemical parameters when farm-workers have not used PPE of their own and after their use (Table 5).

**Table 5:** Hematological and biochemical parameters comparison among farm-workers not using and after their use of PPE.

**Hematological/biochemical parameter (Reference values)**	**Without using any PPE (*n =* 60)**	**After using only commercially available PPE (*n =* 60)**	**Without using any PPE (*n =* 60)**	**After using only cost-effective PPE (*n =* 60)**	**Without using any PPE (*n =* 60)**	**After using both commercial and cost-effective PPE (*n =* 60)**
Vitamin A (30–80 μg mL^−1^)	36.365 ± 4.093	35.710 ± 5.689	35.842 ± 3.671	33.088 ± 4.838	36.365 ± 4.093	36.772 ± 6.492
*p >* 0.01		***p <*** 0.01^**^		*p >* 0.01	
Vitamin E (5.5–17 μg mL^−1^)	7.753 ± 0.721	7.7057 ± 0.66	7.933 ± 0.714	7.846 ± 0.656	7.753 ± 0.721	7.936 ± 0.526
*p >* 0.01		*p >* 0.01		*p >* 0.01	
CRP (1,886 ng mL^−1^)	2,029 ± 344	1,641 ± 133	2,002 ± 335	1,813 ± 130	2,029 ± 344	1,791 ± 91.333
***p <*** 0.01^**^		***p <*** 0.01^**^		***p <*** 0.01^**^	
IL-6 (4.5 pg mL^−1^)	5.1018 ± 0.265	2.923 ± 0.63	4.92 ± 0.513	2.909 ± 0.558	5.018 ± 0.265	2.902 ± 0.517
***p <*** 0.01^**^		***p <*** 0.01^**^		***p <*** 0.01^**^	
IL-1β (pg mL^−1^)	6.387 ± 0.712	5.483 ± 0.602	6.435 ± 0.69	5.447 ± 0.62	6.387 ± 0.712	5.543 ± 0.610
***p <*** 0.01^**^		***p <*** 0.01^**^		***p <*** 0.01^**^	
TNF-α (0.075 pg mL^−1^)	0.096 ± 0.345	0.037 ± 0.021	0.09 ± 0.019	0.033 ± 0.025	0.096 ± 0.345	0.044 ± 0.047
***p <*** 0.01^**^		***p <*** 0.01^**^		***p <*** 0.01^**^	
Cortisol (3.73 pmol mL^−1^)	4.056 ± 0.38	3.707 ± 0.343	3.947 ± 0.433	3.675 ± 0.29	4.056 ± 0.38	3.652 ± 0.34
***p <*** 0.01^**^		***p <*** 0.01^**^		***p <*** 0.01^**^	
AST (0–37 U/L)	28.65 ± 2.673	22.783 ± 1.290	27.783 ± 2.894	23.483 ± 2.00	28.65 ± 2.673	23.23 ± 1.769
***p <*** 0.01^**^		***p <*** 0.01^**^		***p <*** 0.01^**^	
ALT (0–40 U/L)	29.70 ± 2.324	20.917 ± 3.519	30.05 ± 2.332	20.72 ± 3.279	29.70 ± 2.324	20.95 ± 3.605
***p <*** 0.01^**^		***p <*** 0.01^**^		***p <*** 0.01^**^	
Total protein (6–8.3 g dL^−1^)	6.574 ± 1.083	7.013 ± 0.736	6.472 ± 0.971	6.936 ± 0.612	6.574 ± 1.083	7.148 ± 0.568
***p <*** 0.01^**^		***p <*** 0.05^*^		***p <*** 0.01^**^	
A/G ratio (1.2–2.2)	1.544 ± 0.211	1.616 ± 0.268	1.604 ± 0.214	1.561 ± 0.244	1.544 ± 0.211	1.637 ± 0.192
*p >* 0.01	*p >* 0.01	***p <*** 0.05^*^	

Values indicate mean ± standard deviation; Statistical significant differences was considered at

* *p* < 0.05

and

** *p* < 0.01.

#### 3.4.1. Nutrient levels

**Figure 6:**
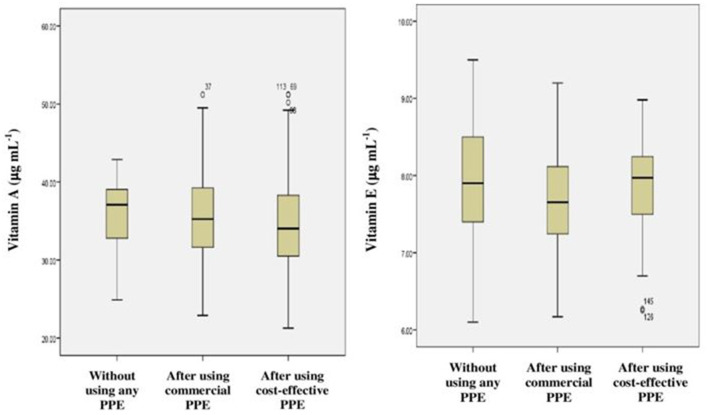
Frequency distribution (Box-plots) for nutrients among farm-workers not using and after their use of PPE.

It was found that the levels of nutrients such as vitamins A and E were low or marginal among all the farm-workers irrespective of the use/non-use of PPE. Hence, no difference was found between their levels among the farm-workers before and after using PPE (Table 5 and Figure 6).

#### 3.4.2. Inflammatory markers

**Figure 7:**
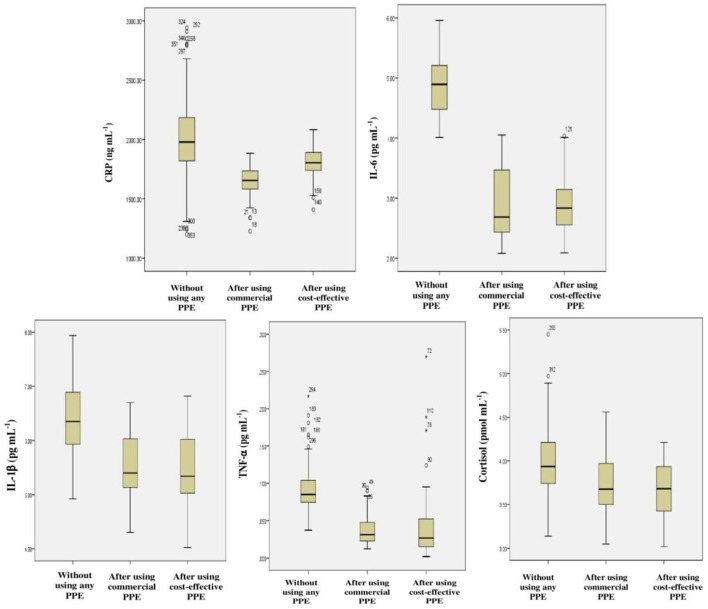
Frequency distribution for various inflammatory markers among farm-workers not using and after their use of PPE.

Increased levels of inflammatory markers such as CRP, IL-6, IL-1β, TNF-α and cortisol were observed among the farm-workers when they have not used PPE as compared to their respective normal values. However, no inflammation was observed among those who have used PPE, showing significant difference in results when compared to those who have not used PPE (*p* < 0.01) (Table 5 and Figure 7).

#### 3.4.3. Liver function tests

**Figure 8:**
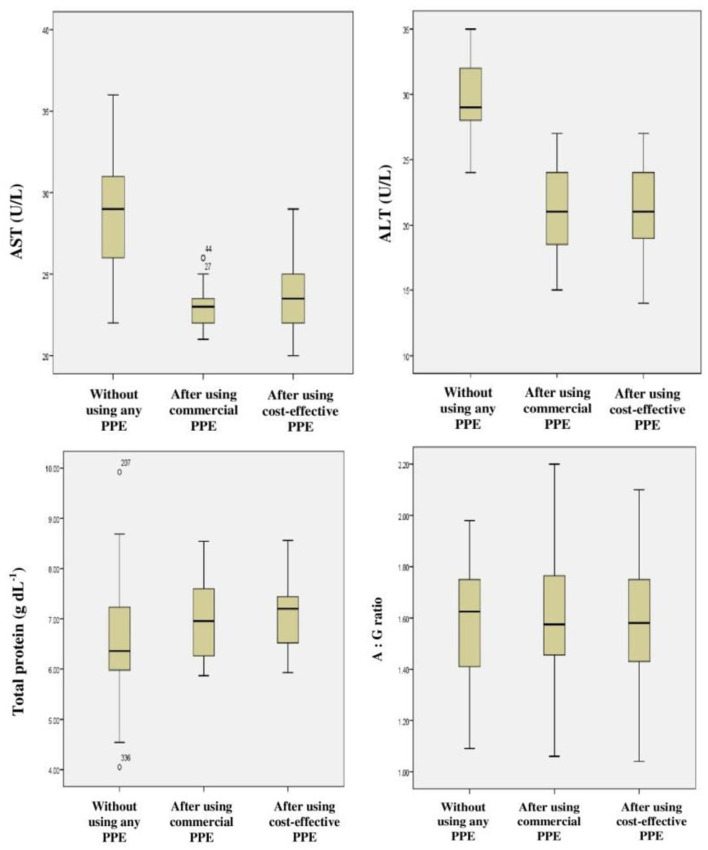
Frequency distribution for liver function tests among farm-workers not using and after their use of PPE.

Hepato-toxicity was monitored by quantitative analysis of the AST, ALT, total protein, and A/G ratio which was used as the biochemical markers for alterations in the liver function. It was found that the liver function profile of the farm-workers was found to be in the normal range; however the levels of AST, ALT, and total protein were significantly altered (*p* < 0.01 and *p* < 0.05) among the subjects after the use of PPE of any type that was provided to them in comparison with those who have not used PPE (Table 5 and Figure 8).

## 4. Discussion

Studies have emphasized that Indian farm-workers who unsafely handle pesticides are at high risk for pesticides associated adverse effects which include AChE inhibition, oxidative stress, hepatic dysfunction, and inflammation (3, 49–52). Therefore, it was indispensable to investigate the problem lying with pesticides exposure and if possible to mitigate the same by suitable means/methods. Although several studies have stressed that the adoption of GAPs and the use of PPE are the ideal methods to minimize the risk of exposure to pesticides among farm-workers, the role of PPE in the minimization of the toxicological effects has not been addressed so far (53, 54). Therefore, the present investigation was designed and conducted among the Indian farm-workers to assess the impact of use of PPE on pesticides exposure and its related toxicity. The present study findings indicate unsafe pesticide handling practices and an insufficient level of risk perception which resulted in the increased levels of biomarkers among farm-workers exposed to pesticides. Linear regression analysis indicates effect of duration of pesticides exposure on AChE activity and levels of various inflammatory biomarkers among exposed farm-workers. However, there was no effect of duration of pesticides exposure on the levels of vitamins A, E, ALT, AST, total protein and A/G ratio. In addition, the findings of present interventional study also revealed a reduction in the levels of biomarkers among farm-workers after the use of PPE (commercially available and cost-effective) provided to them for 90 days when compared to those who have not provided/used PPE.

Questionnaire data and field observations of the present investigation identified several likely risk factors among the farm-workers such as inadequate personal protection during pesticides use, inappropriate clothing, while during handling of pesticides, bare-hand mixing of the pesticide formulations, spraying using damaged equipment, working in tropical climatic conditions and against the wind directions, lack of technical knowledge on hazards of pesticide toxicity, malnutrition, and their reluctance/ignorance to obey GAPs, etc. These risk factors could be responsible either for increase in the likelihood of exposure at higher levels or the toxic effects, however, these are in accordance with data of previous studies conducted in other developing countries (2, 15, 55, 56). Further, farm-workers were also spraying mixtures of pesticides which not only foster occupational exposure but also cause damage to various organs of the body by exhibiting synergistic effects (5, 53, 57). Moreover, the use of potential carcinogens, belonging to moderately (class II) and extremely/highly hazardous (class Ia/Ib) class of pesticides and repeated exposure to elsewhere banned pesticides by the farm-workers is also an issue of concern (58). Additionally, 99% of farm-workers of the present investigation were not using PPE while handling the pesticides, resulting in the entry of pesticides through inhalation and dermal contact (59, 60). Moreover, the self-reported morbidity symptoms made by the farm-workers were similar to those reported among pesticide applicators from other countries (16, 61).

It was evident from the findings of the present study that the increase in AChE inhibition and inflammation found among farm-workers with the increase in the duration of exposure to pesticides when they have not used PPE, is in line with studies reported earlier (5, 53, 62–64). In the present investigation, the AChE inhibition among the farm-workers could be due to the usage of both OPs and CMs pesticides, as they are cholinesterase-inhibiting chemicals that are likely to induce neurotoxic effects (49, 53, 65), when get accumulated in the nerve synapses, which leads to an over-activation of the brain and muscular tissue (66), which could have lead to morbidity symptoms such as headache, backache, weakness and muscle aches, as reported by farm workers in the present study as well. Further, this interventional study also revealed relatively lower inhibition of AChE when they have used both the types of PPE provided to them. This result is in agreement with a study that demonstrated significantly lower AChE activity among farmers who did not use PPE than those using PPE (67).

A plethora of studies showed that micronutrients and vitamins play a pivotal role as antioxidants in regulating the enzymes associated with oxidative stress induced due to environmental contaminants (9, 52, 68). In the present study, the lower levels of vitamins A and E detected among farm-workers could be due to their utilization in scavenging the free radicals generated during the process of oxidation or peroxidation induced due to pesticide residues (2, 52).

Pesticides exert an immunomodulatory effect by inducing macrophage activation and over-expression of inflammatory cytokines viz., TNF-α, IL-1β, and IL-6 (63, 69, 70). The present study also reported higher inflammation in the form of increased in levels of IL-6, IL-1β, TNF-α, and cortisol among farm-workers when they have not used PPE, which are in agreement with studies conducted earlier (2, 50). Further, the increased CRP levels among farm-workers could also be due to an outcome of acute injury or due to the onset of inflammation in the liver (55, 65). Results of present study revealed that adequate use of PPE has significantly minimized the inflammation (*p* < 0.01). Similar results were also suggested earlier by Madani and co-workers (2) who showed that the adoption of personal protection by the farmer during pesticide handling results in reduction of inflammation.

The liver plays an important role in maintaining the body homeostasis through various processes including metabolism and detoxification of drugs and xenobiotics (12, 71). The long term exposure to pesticides is known to exert adverse effects on total proteins, albumin, urea, ALT, AST, and ALP, resulting in hepatic toxicity and nephro-toxicity (2). In the present study, slightly high levels of AST and ALT were observed among farm-workers when not using PPE as compared to those using PPE. This is in agreement with findings of other studies conducted earlier (5, 65, 67). The results of the present study also revealed that the levels of total protein and A/G ratio were in the normal range and are in accordance with studies reported previously. It is assumed that this normal value could be due to intake of non-vegetarian diet by farm-workers (65, 72).

The limitation of this study is that the farm-workers were exposed to a complex and variable mixtures of substances, some of which have either anti-mutagenic activity or may interact synergistically making it difficult to identify the cause for adverse effects due to a particular agent/chemical. Further, in the present study, the baseline data on the enzyme levels before their exposure to chemicals were also not obtained because of their continuous participation in the farming activities. Another possible limitation of the current study is that their potential exposure to UV rays, weeds, pollens, etc. which might influence inflammatory and other markers. However, an in-depth research is needed to study the same.

## 5. Conclusion

To the best of our knowledge, a few epidemiological studies have assessed the toxicological levels of exposure among the occupationally exposed Indian farm-workers using PPE and comparative studies with those not using PPE. Hardly, any such studies are available to assess the impact of use of either commercially available (which are not in the reachable range of the farmers) and/or the cost-effective PPE (prepared using the available resources) and on the vital biomarkers of pesticides associated toxicity and adverse health effects such as AChE inhibition, enhanced inflammation, and alteration in the liver function tests and other biochemical impairments followed by non-adoption of the GAPs and lack of awareness on protective measures. The current interventional study demonstrates a reduction in the levels of crucial biomarkers among farm-workers who used PPE provided to them for 90 days in any form (commercially available and cost-effective) when compared to those who have not provided/used PPE. This study also suggests that duration of pesticides exposure has profound effect on AChE activity and levels of various inflammatory biomarkers among farm-workers exposed to pesticides. Taken together, this study demonstrated the importance of the use of PPE during pesticide applications and other agricultural tasks to minimize pesticide-associated adverse health effects. Future research is required to scale-up the important findings / leads generated from this study for conducting further intervention studies in other geographical areas of the country and design/develop indigenously, affordable and reusable PPE to reduce the higher risk of adverse health effects among occupationally exposed farm-workers.

## Data availability statement

The raw data supporting the conclusions of this article will be made available by the authors, without undue reservation.

## Ethics statement

The studies involving human participants were reviewed and approved by the Ethical Committee of the Indian Council of Medical Research—National Institute of Nutrition, Hyderabad, India (REF NIN Protocol Number 11/I/2016). The participants provided their written informed consent to participate in this study.

## Author contributions

SL participated in the analysis of the data and interpretation of the results, and wrote the first draft of the manuscript. PJ contributed to the conception and design of the study and wrote sections of the manuscript and edited whole manuscript to bring it to present form. BJ reviewed and edited first draft of manuscript, provided critical inputs on data analysis and data interpretation using an appropriate statistical analysis. PY, AP, JV, and MN were involved in the fieldwork and collection and processing of samples. BK has done the statistical analysis. All authors contributed to manuscript revision, read, and approved the submitted version.

## Supplementary material

Supplementary File Questionnaire administered to the farm-workers.
